# Impact of COVID-19 on a urology residency program

**DOI:** 10.1590/S1677-5538.IBJU.2020.0707

**Published:** 2021-02-03

**Authors:** Alexandre Danilovic, Fabio Cesar Miranda Torricelli, Gabriel dos Anjos, Mauricio Dener Cordeiro, Marcos Giannetti Machado, Miguel Srougi, William C. Nahas

**Affiliations:** 1 Universidade de São Paulo Faculdade de Medicina Hospital das Clínicas São Paulo SP Brasil Departamento de Urologia, Hospital das Clínicas, Faculdade de Medicina da Universidade de São Paulo, São Paulo, SP, Brasil.; 2 Universidade de São Paulo Faculdade de Medicina Hospital das Clínicas São Paulo SP Brasil Divisão de Urologia, Hospital das Clínicas, Faculdade de Medicina da Universidade de São Paulo, São Paulo, SP, Brasil.

## INTRODUCTION

The coronavirus disease 2019 (COVID-19) pandemic emerged in China and has rapidly spread worldwide taking advantage of growing commerce, financial, and social connectivity to the epicenter of the outbreak (1, 2). The novel SARS-CoV-2, an enveloped RNA virus from the coronaviruses family, was identified as the cause of COVID-19 (3). The disease is a viral respiratory infection with a variable systemic compromise. To date, no specific treatment or vaccine exists, imposing a policy of limiting exposure to reduce virus spread (4). Although the majority of ill patients do not need hospital care, as much as 5% require intensive care for days until weeks (5). The huge numbers of patients seeking medical attention at the same time and monopolizing hospital facilities caused a shift in the healthcare system's focus towards critically ill patients (6–8).

The COVID-19 pandemic has imposed a new reality to urology residency worldwide (9, 10). Surgical procedures were reduced to urgent care causing an abrupt decrease in surgical volume. Visits to the medical office and physical examination were also reduced to a minimum to ensure patients' and staff safety. Academic curriculum previously based on classroom lessons, group discussion and departmental meetings were no longer possible due to commitment to social distancing. Specialized staff members were converted to COVID-19 care physicians (11, 12). Disruption of previous urology residency programs occurred at all levels.

Professional formation of urology residents who face this new reality had to be adapted to mitigate the loss of surgical skills and knowledge. Our hypothesis is the shift in focus from the hospital to care for critically ill patients with COVID-19 may have reduced the surgical volume of residents. This study aimed to critically analyze the impact of COVID-19 on surgical volume and academic activities of a urology residency program in an Academic Center.

## MATERIAL AND METHODS

This study was approved by the Research Ethics Committee of the University of Sao Paulo School of Medicine (project number CAPPESQ 15398/2020). The Urology Department of our Institution has eighteen residents. Six residents are admitted per year for a three-year course after a two-year training in general surgery. Electronic records of surgeries performed by all urology residents from the third, fourth and fifth years of residency assisted by staff members from February 27^th^ to May 30^th^, 2017, 2018, 2019, and 2020 were analyzed. All surgeries performed in hospitalized patients were included. Outpatient, office-based and ambulatory, procedures such as cystoscopy and biopsy were excluded from this study.

Surgeries were categorized according to complexity in minor (e.g. surgery on the external genitalia, stent placement), medium (e.g. transurethral resections of the bladder/prostate or ureteroscopy), and major surgeries (e.g. oncological open or laparoscopic/robotic surgery on the prostate, bladder, or kidney) (13). Duration of each type of surgery was categorized to compare estimated hours spent in the operation room (OR) in less than 2 hours (e.g. transurethral resection of the bladder/prostate, ureteral stent placement, retrograde intrarenal surgery and urethroplasty), 2 to 4 hours (e.g. pyeloplasty, percutaneous nephrolithotomy, radical prostatectomy, nephrectomy, kidney transplantation) and more than 4 (e.g. radical cystectomy). The overall number of surgeries, complexity of surgeries, estimate OR time and surgeries per residency year were compared between years. Academic curriculum composed of classroom lessons, group discussion, departmental meeting and online learning was compared between years according to cumulative hours spent on academic activities.

Categorical data were described in frequencies and percentages and compared using the Chi-square test. Continuous data were described as mean and standard deviation and compared using the Student T-test. All statistical analysis was performed using SPSS version 20.0 (SPSS Inc. Chicago, IL, USA). The significance level was set at p <0.05.

## RESULTS

Overall surgical volume significantly decreased in 2020 when compared to the previous three years from February 27th to May 30th (p <0.001). The surgical volume in 2020 was reduced by 50.8% when compared to the mean of the last three years. The volume of minor, medium and major complexity surgeries decreased in 2020 when compared to the past three years (p <0.001, p=0.094 and p=0.018, respectively). Surgical volume categorized by estimated time spent in OR decreased in 2020 when compared to the previous three years for surgeries lasting less than two hours, from two to four hours and more than four hours (p <0.001, p <0.001 and p=0.002, respectively). Surgical volume decreased in the third, fourth and fifth year of the residency program (p <0.001, p <0.001and p=0.004). The proportion of surgeries performed by complexity estimated OR time and year of residency remained stable throughout the studied period.

**Figure 1:**
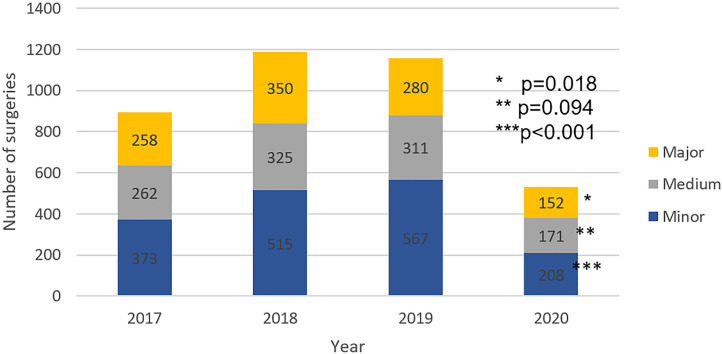
Comparison of surgical volume categorized by complexity from February 27th to May 30^th^.

**Figure 2:**
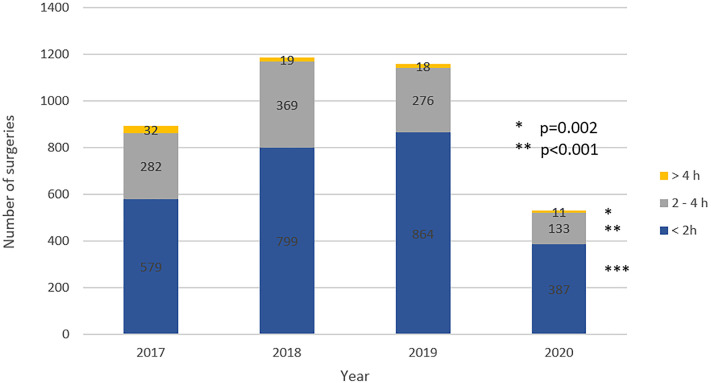
Comparison of surgical volume categorized by OR time from February 27th to May 30^th^.

**Figure 3:**
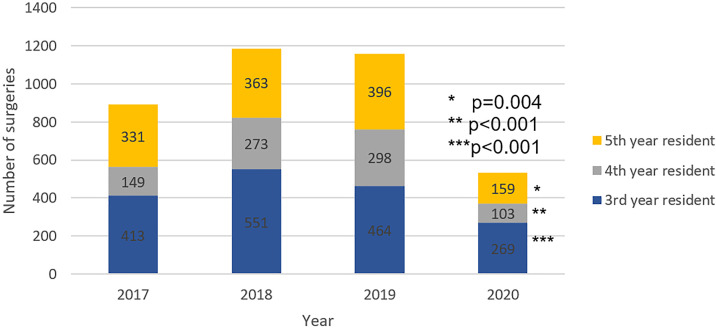
Comparison of surgical volume categorized by year of urology residency from February 27th to May 30^th^.

Reduction of surgical volume in 2020 in comparison to the mean of the three previous three years was 57.0% in minor surgeries, 49.8% in medium surgeries and 48.6% in major surgeries (Figure-1), 48.2% in surgeries with less than 2 hours of OR time, 59.9% in surgeries with two to four hour of OR time and 52.2% in surgeries with more than four hours of OR time (Figure-2), 43.5% for the third-year resident, 57.1% for the fourth year resident and 56.2% for the fifth year resident (Figure-3).

Overall hours spent on academic activities remained stable in 2020 when compared to the previous three years during the same period (122h vs. 121h, p=1).

## DISCUSSION

The COVID-19 pandemic had negatively impacted the volume of surgeries of our urology residency program. Surgical volume decreased in minor, medium and major complex surgeries, in surgeries with less than 2 hours, two to four hours and more than four hours of OR time, and to all residency years, keeping the proportion of previous years. The overall reduction of the surgical volume was 50.8%. The COVID-19 pandemic impacted in similar way surgery from all complexity and all residency years. The number of hours spent on academic activities remained stable.

This study was conducted in a university complex of ten hospitals distributed in 600.000m^2^ in the urban area of the largest city of the American continent with a total of 2.400 hospital beds. The urology residency is composed of two years rotation in the general surgery department plus three years of specific rotation in the urology department. The specific urology residency program has six residents per year. Surgical training, academic activities and laboratory skills are developed in andrology, bladder outlet disorders, reconstructive surgery, urological cancer, urolithiasis and endourology, kidney transplantation and pediatric urology. The first case of COVID-19 in our country was reported on February 27 (14). Since then, one of the hospitals from the complex was converted to an exclusive COVID-19 care center with 300 critical care beds and 300 infirmary beds. Also, the entire complex shifted to prioritize urgent and critical care and postpone elective surgeries and routine consults.

Other urology residency programs from countries affected by the COVID-19 pandemic also reported a severe negative impact on clinical and surgical activities. A survey conducted in Italy reported that up to 81% and 62% of urology residents experienced a severe reduction in clinical training exposure and surgical activities, respectively. Urology residents reported 81.2% reduction of ambulatory visits, 74.2% of diagnostic procedures, 62.1% of endoscopic surgeries, 57.8% of open surgeries and 44.2% of minimally invasive surgeries (15). Sixty percent of urology programs from the United States reported concern that residents will not achieve case minimums due to COVID-19 (16). Medical residents from other specialties also felt that the reduction in surgical volume during this pandemic will negatively impact on their surgical training (17, 18).

The increase of online learning using telemedicine and collaboration with other centers and laboratory skills development are valid tools to reduce loss in academic activities and the volume of surgeries (15). Our residents have access to a simulation laboratory for surgical skills development, where they can train laparoscopic and microscopic procedures in a dry lab and a wet lab with porcine surgeries (19–21). These activities may help to maintain technical skills. Another recommended action is to watch high-quality surgical videos from the online education library of specialty associations (22). Due to the absence of uniformity in the urology training program, it is not possible to propose a standard protocol to mitigate the impact of COVID-19. Rather, urology residency programs should evaluate the local impact of COVID-19, considering the particularities of each program, to fill the specific gap as soon as possible (15, 22). Attempts to mitigate the loss of surgical skills and knowledge of our residents are being made, however, it is not possible at this moment to state if the pandemic effects on urology learning will last and compromise the professional formation. While medical residency programs are struggling to mitigate cognitive medical education, this crisis provides a unique opportunity for non-cognitive development not only for residents but also for staff, including resilience and community awareness (23). Non-cognitive skills may help increase overall satisfaction among medical professionals (24).

The COVID-19 pandemic possibly caused a permanent change in our mindset. A strong public healthcare system is fundamental to assist vulnerable populations with standard medical care to face a health crisis (25). Also, more collaboration among academic hospitals, online teaching and providing care for distant patients are some of the possibilities of telemedicine that came to stay in our daily life as healthcare providers (22).

This study has limitations. It was conducted in a single Institution and may not reflect the same impact of COVID-19 on other urology residencies. Nevertheless, the overall impact of COVID-19 on a University Centre could be evaluated by this study. Also, due to the retrospective method of this study, the hours spent in the operating room were estimated and are not the actual hours spent. However, this is an acceptable flaw because the same method was applied throughout the study, not compromising interpretations. Evaluation of the long-term impact of the COVID-19 pandemic is not possible now. However, urology residency programs have already perceived losses in surgical volume.

## CONCLUSION

The COVID-19 pandemic reduced the surgery volume of our urology residency program in around 50% for all complexity levels of surgery and all years of residency.
